# Molecular Characterization of LRB7 Gene and a Water Channel Protein TIP2 in* Chorispora bungeana*

**DOI:** 10.1155/2016/2483258

**Published:** 2016-09-01

**Authors:** Ming Li, Zhaoxu Liang, Cuixia Di, Weikuan Fang, Kaichao Wu, Maoshan Chen, Shanshan He, Yuan Zeng, Yan Jing, Jun Liang, Fang Tan, Song Li, Tuo Chen, Guangxiu Liu, Lizhe An

**Affiliations:** ^1^Sugarcane Research Institute, Guangxi Academy of Agricultural Sciences, Nanning 530007, China; ^2^Cold and Arid Regions Environmental and Engineering Research Institute, Chinese Academy of Sciences, Lanzhou 730000, China; ^3^Institute of Modern Physics, Chinese Academy of Sciences, Lanzhou 730000, China; ^4^Guangxi Academy of Agricultural Sciences, Nanning 530007, China; ^5^Department of Biochemistry and Genetics, La Trobe Institute for Molecular Science, La Trobe University, Melbourne, VIC 3086, Australia; ^6^State Key Laboratory of Arid Agroecology, Lanzhou University, Lanzhou 730000, China

## Abstract

*Background*. Water channel proteins, also called aquaporins, are integral membrane proteins from major intrinsic protein (MIP) family and involved in several pathways including not only water transport but also cell signaling, reproduction, and photosynthesis. The full cDNA and protein sequences of aquaporin in* Chorispora bungeana* Fisch. & C.A. Mey (*C. bungeana*) are still unknown.* Results*. In this study, PCR and rapid amplification of cDNA ends approaches were used to clone the full cDNA of LRB7 (GenBank accession number: EU636988) of* C. bungeana*. Sequence analysis indicated that it was 1235 bp, which had two introns and encoded a protein of 250 amino acids. Structure analysis revealed that the protein had two conserved NPA motifs, one of which is MIP signature sequence (SGxHxNPAVT), six membrane helix regions, and additional membrane-embedded domains. Phylogenetic analysis suggested that the protein was from TIP2 subgroup. Surprisingly, semiquantitative RT-PCR experiment and western blot analysis showed that LRB7 and TIP2 were only detectable in roots, unlike* Arabidopsis* and* Raphanus*. Connecting with our previous studies, LRB7 was supported to associate with chilling-tolerance in* C. bungeana*.* Conclusion*. This is the first time to characterize the full sequences of LRB7 gene and water channel protein in* C. bungeana*. Our findings contribute to understanding the water transports in plants under low temperatures.

## 1. Introduction

Water channel proteins (aquaporins) are integral membrane proteins from a large family of major intrinsic protein (MIP) [1]. The discovery of first water channel protein (AQP1) in human erythrocytes explained the high water permeability of red blood cells and renal tubular epithelial cells [2]. Since the first plant-related water channel protein was found in* Arabidopsis thaliana* called *γ*-tonoplast intrinsic protein (*γ*-TIP, TIP1) [3], hundreds of water channel proteins have been identified in kinds of organisms including bacterial, animals, plants, and human [4–7]. In plants, aquaporins were classified into five subgroups including plasma membrane intrinsic protein (PIP) [8], tonoplast intrinsic protein (TIP) [9], nodulin-26 like intrinsic protein (NIP) [10], small basic intrinsic protein (SIP) [11], and X intrinsic protein (XIP) [12]. The structure of aquaporins is highly conserved, containing six transmembrane helices and two additional membrane-embedded domains linked by five loops, with the C- and N-terminals which are located on the cytoplasmic surface of the membrane [13, 14]. Aquaporins have been reported to be involved in root water transport, cell signaling, reproduction, and photosynthesis [15, 16].

*Chorispora bungeana* Fisch. and C.A. Mey. (*Chorispora bungeana*,* C. bungeana*) is an alpine perennial herb belonging to the Brassicaceae family. It grows in the freezing and thawing tundra in the borders of glaciers, subalpine meadow, and gravel. Some studies of this model plant have investigated mainly cold-tolerance [17–19]; however, the mechanism of water transport in freezing environment is asking for more experiments and evidence. In* Arabidopsis* and* Raphanus*, aquaporins have been characterized with high expression in young shoot, rosette, and flower tissues and other tissues [20, 21]. In this study, we first isolated the* LRB7* cDNA sequence encoding an aquaporin in the root of* C. bungeana*. In addition, the encoding aquaporin was characterized as *δ*-TIP, also called TIP2 [22]. We further found LRB7 and its protein product TIP2 probably detectable only in the root tissue, not in the stems or leaves of* C. bungeana*.

## 2. Materials and Methods

### 2.1. Sample Collection

The wild species of* C. bungeana* were grown in the source area of Urumqi River in Tianshan Mountains, Xinjiang, China (43°05′ N, 86°49′ E, with an altitude of 3,800–3,900 m), and transplanted to the experimental field of botanical garden at Lanzhou University. After one year, leaves, stems, and young roots were collected from three individual plants during their growth period (from June to September) and stored in liquid nitrogen.

### 2.2. DNA Extraction

Genomic DNA was isolated according to the method described by Doyle and Dickson [23]. Young root samples (200 mg) were grinded in mortar and pestle in 10 mL of 2 × CTAB isolation buffer (2 × CTAB = 2% hexadecyltrimethylammonium bromide (Sigma-Aldrich), 100 mM Tris-HCl pH 8.0, 1.4 M NaCl, 20 mM EDTA, and 0.2% 2-mercaptoethanol). Then the CTAB/plant extract mixture was incubated at 60°C for 1 hr in a recirculating water bath and centrifuged at 12000 ×g for 5 min to remove cell debris. The supernatant was transferred to a clean microfuge tube, added in 250 *µ*L of chloroform : isoamyl alcohol (24 : 1), mixed by inversion, and centrifuged at 13000 RPM for 1 min. The supernatant (contains the DNA) was next moved to a clean tube and added 50 *µ*L of 7.5 M ammonium acetate followed by 500 *µ*L of ice-cold absolute ethanol. To precipitate the DNA, invert the tubes slowly several times and incubate the tubes at −20°C for 1 hr. After precipitation, samples were centrifuged at 7000 RPM for 1-2 min to concentrate the DNA. The pellet was then washed by 20 mL of wash buffer (70% ethanol and 10 mM ammonium acetate): following an initial gentle swirling of the tube to break up the pellet, samples were left at room temperature for 20 min. The DNAs were then centrifuged at 13000 RPM in for 5 min, air-dry the pellets (~30 min), were resuspended in 1 mL TE (10 mM Tris-HC1, 1 mM EDTA, pH 7.4), and were incubated in RNase A solution (10 *µ*g, Sigma-Aldrich) for 30 min at 37°C. The solution was made 2.5 M in ammonium acetate and 2.5 volumes of cold ethanol were added to precipitate DNA. The DNA pellet was then air-dried again on a desktop overnight and resuspended in 1 mL TE. The quality of DNA was evaluated and controlled by NanoDrop 2000 (Thermo Scientific).

### 2.3. Total RNA Isolation

Total RNA was isolated using Trizol® reagent (Invitrogen) according to the manufacture's instruction. Briefly, a total of 200 mg plant samples (young roots, leaves, and stem samples) were incubated in 2 mL Trizol reagent at room temperature for 5 min. After transferring the mixture into clean tubes and adding 0.4 mL of chloroform, the samples were homogenized by shaking the tubes for 15 sec, incubated for 3 min, and centrifuged at 12,000 ×g for 15 min at 4°C. The aqueous phase was transferred into new tubes. RNase-free glycogen (5 *µ*g) was added in as carriers, followed by 1 mL of 100% isopropanol. Next, the samples were incubated at room temperature for 10 min and centrifuged at 12,000 ×g for 10 min at 4°C. The RNA pellet was washed by using 2 mL of 75% ethanol, vortex briefly, and centrifugation at 7500 ×g for 5 min at 4°C. The RNA pellet was air-dried, suspended in RNase-free water, water-bathed at 57°C for 10 min, and stored at −80°C. RNA quality was evaluated by Agilent 2100 Bioanalyzer. Young roots, stems, and leaves were used for RNA isolation separately.

### 2.4. RT-PCR

**Figure 1:**
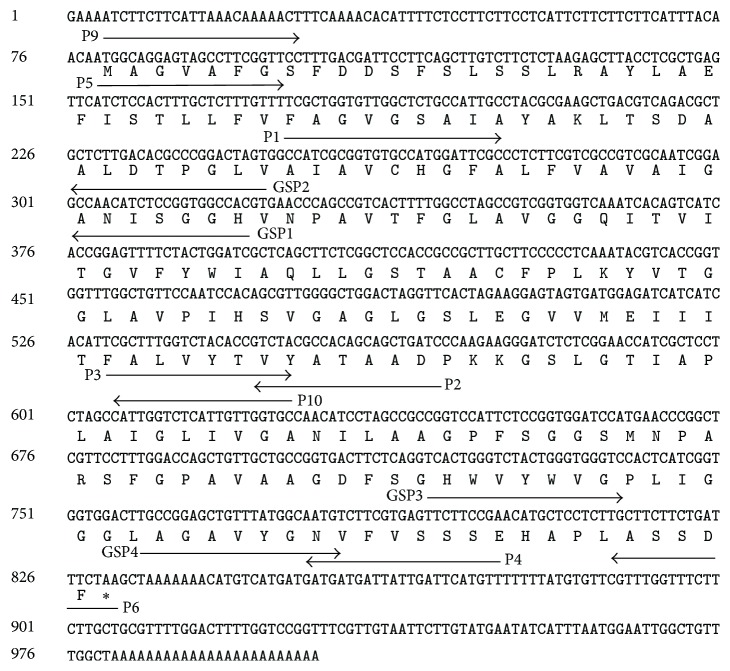
LRB7 cDNA sequence, deduced amino acid sequence, and PCR primer sequences (underlined by black line with arrow).

To confirm the full sequence of LRB7 gene, PCR amplification was performed using primers shown in Table 1 and Figure 1. Total RNA (2 *µ*g) was used for cDNA synthesis with One Step RNA PCR Kit (Takara Bio) and AMV Reverse Transcriptase XL (Takara Bio), according to the protocols. The reaction mixture contained 1.25 U of Ex Taq DNA polymerase (Takara Bio), 2.5 *μ*L of 10x buffer (100 mM Tris-HCl (pH 8.3), 500 mM KCl, 15 mM MgCl_2_), 2.5 mM of each dNTP, and 0.4 *μ*M of each primer. PCR amplifications were performed at 94°C for 5 min followed by 30 cycles of amplification (94°C for 30 s, 59°C for 30 s, and 72°C for 30 s) and a final elongation at 72°C for 5 min.

### 2.5. 5′ and 3′ RACE Experiment and DNA Sequencing

The reactions of Rapid Amplification of cDNA Ends (RACE) were performed using GeneRacer*™* RACE Ready cDNA Kit (Invitrogen) strictly following the manufacturer's instructions. The amplified fragments were then transferred into the pMD18-T vector (Takara Bio), nucleotide sequences were determined with CEQ 2000XLDNA Analyzer (Beckman Coulter), and the data were analyzed and visualized by using Sequencer (Genes Codes Corporation). Three independent clones of each amplification product were sequenced to avoid errors caused by PCR. The primers used for this experiment can be found in Table 1.

### 2.6. Semiquantitative RT-PCR

To analyze the tissue specificity of LRB7 at transcriptional level in* C. bungeana*, semiquantitative RT-PCR experiments were conducted using the RNA samples isolated from roots, stems, and leaves, respectively. Briefly, total RNA (2 *µ*g) was used for cDNA synthesis, as described. Primers P7/P8 and P9/P10 (Table 1) were designed for the housekeeping* actin* gene (GenBank accession number AY825362) and LRB7 gene determined in this study. PCR amplifications were performed following the process: 25 cycles (94°C for 5 min), 30 cycles (94°C for 30 s, 50°C for 45 s, and 72°C for 30 s), and a final elongation at 72°C for 10 min. The PCR products were separated and purified using 1% agarose gels and stained with ethidium bromide and analyzed by Gene Tools software (Gene Company Ltd.).

### 2.7. Preparation of Antibodies for LRB7 Encoded Protein (Anti-LRB7)

pGEX-4T-3-LRB7 plasmid was built by combining LRB7 coding regions and PGEX-4T-3 plasmid and introduced into* E. coli* BL21 (DE3) pLysS. The transformed* E. coli* BL21 (DE3) pLysS was cultured in lysogeny broth (LB) medium for overnight. Then, 0.4 mL of liquid bacteria was cultured in 20 mL LB medium until the optical density (OD) reached 0.6 at 600 nm. By adding 24 *µ*L of 20% isopropyl-*β*-D-thiogalactoside (20% IPTG), fusion proteins (GST-LRB7) were induced for 6 hr from 1 liter of liquid transformed bacteria, obtained by boiling for 12 min, separated by 12% SDS-PAGE gels, and purified by using GSTrap 4B (GE Healthcare Life Sciences) following the protocols. Next, purified GST-LRB7 proteins (100 *µ*g) and Complete Freund's Adjuvant (equal volume to GST-LRB7 protein medium, Sigma-Aldrich) were used to simultaneously immunize two Japanese adult male rabbits every two weeks. Blood serum of the rabbits was harvested and polyclonal antibody was purified by Protein A Sepharose (CL-4B) following standard protocol (Invitrogen) and purified by using affiliation column with bound GST to remove the anti-GST antibody. The purified anti-LRB7 was stored at −80°C in a buffer containing 1% BSA, 50% glycerol, and 0.02% sodium azide for further use.

### 2.8. Western Blot Analysis

Plant samples (200 mg) of roots, stems, and leaves were lysed by using Radio Immunoprecipitation Assay (RIPA lysis buffer) following protocol (Beyotime Institute of Biotechnology) and homogenized in the ice-cold RIPA lysis buffer (150 mM sodium chloride, 1% NP-40, 0.5% sodium deoxycholate, 0.1% SDS, 50 mM Tris-HCl pH 8.0, and a mixture of protease inhibitors (Applygen Technologies Inc.)). After the precipitate was discarded, crude membrane fraction was collected by centrifugation at 12,000 RPM for 20 min at 4°C. Protein samples (30 *µ*g) were separated by 12% SDS-PAGE gels and then blotted onto PVDF membrane (EMD Millipore) following the standard protocol. Primary antibody (anti-LRB7) was used to detect TIP, followed by secondary antibody (goat-anti-rabbit IgG, Applygen Technologies Inc.). The signals were detected and visualized by using ECL Western Blotting system (Amersham Bioscience).

### 2.9. Bioinformatics Analysis

The deduced protein of LRB7 gene was aligned with TIPs from other species using BioEdit 7.2.5 (http://www.mbio.ncsu.edu/bioedit/bioedit.html). The phylogenetic tree was constructed based on the genetic distance of protein sequence by ClustalX2 [24] and visualized by MEGA6 software (http://www.megasoftware.net/). The transmembrane helices of TIP2 were predicted using an online software TMHMM 2.0 (http://www.cbs.dtu.dk/services/TMHMM-2.0/). The membrane-spanning regions and its orientation were predicted using TMpred (http://www.ch.embnet.org/software/TMPRED_form.html).

## 3. Results

### 3.1. Characterization of LRB7 Gene in* C. bungeana*

To characterize the full cDNA sequence of LRB7 in* C. bungeana*, RB7 gene sequences from other species including* Helianthus annuus* (GenBank accession number X95953),* Nicotiana tabacum* (GenBank accession number S45406),* Lycopersicon esculentum* (GenBank accession number LEU95008), and* Daucus carota* (GenBank accession number AB000506) were used to design PCR primers P1, P2, and P4 (Figure 1). Using P1 and P2 primers, we initially obtained 392 bp fragment from the* C. bungeana* cDNA. Then, we used the resulting cDNA fragment to design P3 primer (Figure 1), obtained a fragment of 271 bp sequence by using P3 and P4 primers, and assembled a longer fragment (~626 bp) together with the first fragment. Further, based on this ~626 bp sequence we predicted the primers of 5′ GSP1, 5′ GSP2, 3′ GSP3, and 3′ GSP4 (Figure 1) for 5′ and 3′ RACE experiments and obtained the full length LRB7 cDNA (1004 bp, accession number EU636988) in* C. bungeana*. Then the full cDNA of LRB7 was used to design primers of P5 and P6 (Figure 1), which were used for* C. bungeana* genomic DNA amplifications. It is not surprising that the DNA sequence (958 bp) is consistent with the LRB7 cDNA sequence characterized by the 5′ and 3′ RACE experiments.

**Figure 2:**
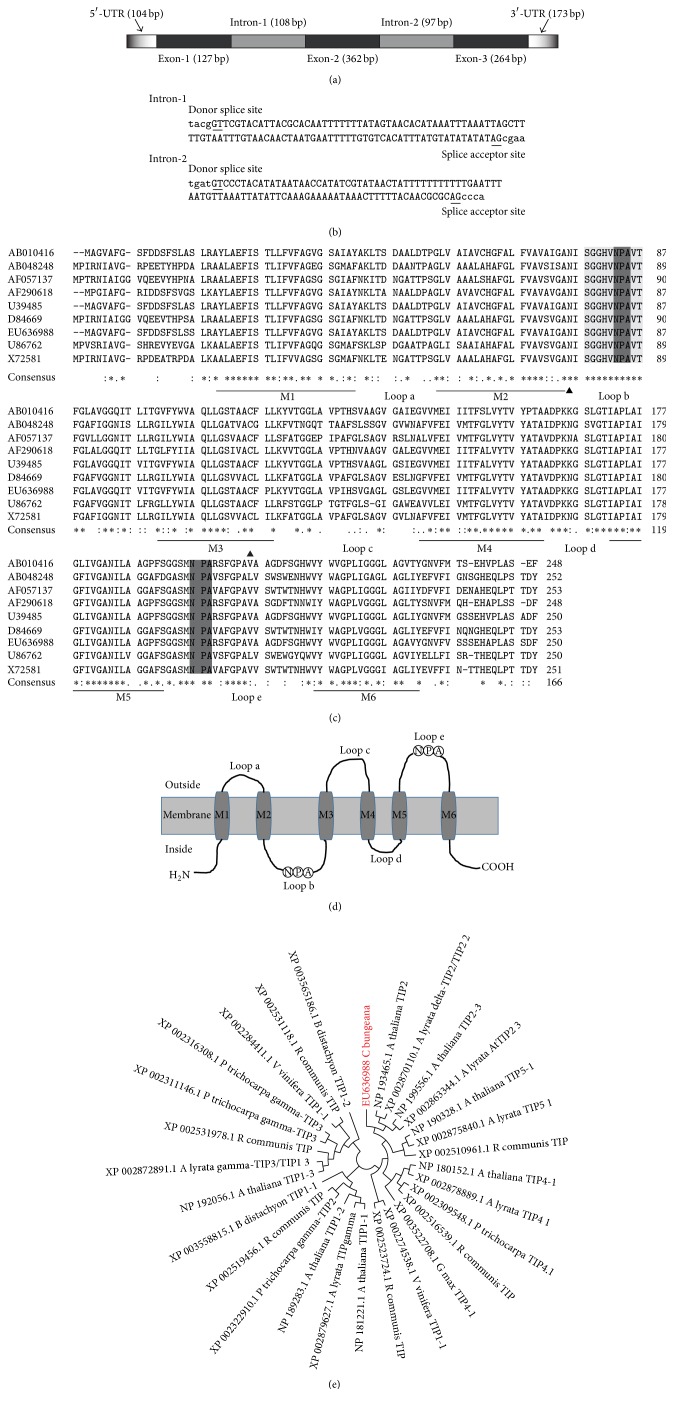
Structures and phylogenetic analysis of LRB7 gene and TIP2. (a) Gene structure of LRB7 showed that two introns can be identified. (b) Both intron-1 and intron-2 satisfied the GT/AG splicing rule. (c) Alignment of deduced amino acid sequence of LRB7 and other 8 TIPs showed two conserved NPA motifs (loop b and loop e) of which loop b was proved to be MIP signature sequence (SGxHxNPAVT), N-glycosylation site (first black triangle arrow), and mercury-sensitive site (cysteine residue, second black triangle arrow). The TIPs are from* Arabidopsis thaliana* (X72581, AF057137, and U39485),* Raphanus sativus* (D84669 and AB010416),* Pyrus communis* (AB048248),* Triticum aestivum* (U86762),* Chorispora bungeana* (EU636988), and* Nicotiana glauca* (AB010416). Using TMHMM-2.0, we predicted six transmembrane helix regions (M1 to M6, underlined) in the protein of LRB7. Its topography in the vacuolar membrane (d) showed high consistence with the model drawn by Daniels et al. [20]. (e) Phylogenetic analysis of 29 TIP sequences told us that the protein encoded by LRB7 in* C. bungeana* was TIP2 (in red).

Next, the cDNA sequence of LRB7 in* C. bungeana* was identified to contain an open reading frame (ORF) of 753 bp, 5′-untranslated region (5′-UTR, 104 bp), 3′-untranslated region (3′-UTR, 173 bp), and 24 bp poly(A) tail (Figure 1). By comparing the cDNA sequence to genomic sequence, we identified three introns in LRB7 gene sequence, shown in Figure 2(a). The AT content of intron-1 and intron-2 was 84% and 75%, respectively. While analyzing the splicing sites, both introns conformed the standard GT/AG rule (Figure 2(b)).

### 3.2. Characterization of the Protein Encoded by LRB7 in* C. bungeana*

Then we characterized the protein product of LRB7 in* C. bungeana*. LRB7 was predicted to encode a protein of 250 amino acids, shown in Figure 1. The alignment of the deduced protein and eight other TIP sequences from species inducing* Arabidopsis thaliana*,* Raphanus sativus*,* Pyrus communis*,* Triticum aestivum*,* Nicotiana glauca*,* Populus trichocarpa*,* Tamarix albiflonum*,* Oryza sativa*, and* Saccharum officinarum* showed that the deduced protein had two conserved characteristic motifs of asparagines-praline-alanine (NPA) (Figure 2(c)). The first NPA motif, which is MIP family marker sequence (SGxHxNPAVT) [25], was found in loop* b* and the second NPA motif was found in loop* e*. Furthermore, six transmembrane helix regions (M1–M6) together with the N- and C-terminals towards the cytoplasmic side were predicted to strongly support that the protein product of LRB7 was from MIP (Figure 2(d)) family.

We next used ClustalX2 to analyze the conservation between the protein encoded by LRB7 and other MIP sequences. In total, we obtained 119 MIP sequences of 13 other species from NCBI database (Table 2). Phylogenetic tree analysis showed that LRB7 encoded aquaporin in* C. bungeana* was from TIP subgroup (see Figure S1 in Supplementary Material available online at http://dx.doi.org/10.1155/2016/2483258). We showed the phylogenetic tree analysis of LRB7 encoded protein with TIP sequences from other species (Figure 2(e)). These results told us that LRB7 encoded protein in* C. bungeana* was TIP2 [26–29]. In addition, the putative TIP2 encoded by LRB7 showed identity of 95.6%, 61%, and 36% with TIP2 (AY085921) from* A. thaliana*, TIP1 (D84669) from* R. sativus*, and PIP1 (EU585602) from* S. officinarum*, respectively.

### 3.3. LRB7 Gene Expression in Roots, Stems, and Leaves of* C. bungeana*

**Figure 3:**
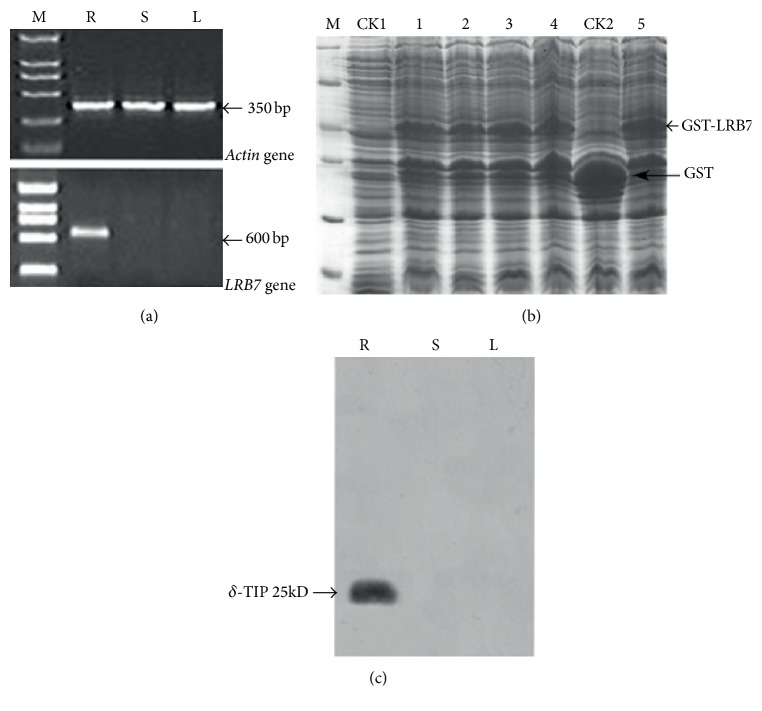
LRB7 expression and TIP2 abundance in leaves (L), stems (S), and roots (R) of* C. bungeana*. (a) Semiquantitative RT-PCR showed LRB7 expressed exclusively in roots, M: DL2000 marker. (b) Quantitative experiment for total proteins from the cell lysates of* E. coli* BL21 (DE3) pLysS transformed with PGEX-4T-3-LRB7. M: unstained protein molecular weight marker; CK1: cell lysates without induction with 20% IPTG; CK2: cell lysates inducted with 20% IPTG; 1–5: total proteins of the cell lysates of* E. coli* BL21 (DE3, induced by 20% IPTG) cultured at 2, 3, 4, 5, and 6 hr, respectively. (c) Western blot analysis of TIP2. The anti-LRB7 specifically recognized 25 kD band in* C. bungeana* roots (R), but not in stems (S) or leaves (L).

We next examined the expression of LRB7 gene in different tissues of* C. bungeana* by semiquantitative RT-PCR. The* actin* gene was used as internal control and primers for* actin* and LRB7 can be found in Table 1. Interestingly, the results showed that LRB7 gene was exclusively detectable in roots. In Figure 3(a), roots, stems, and leaves shared consensus PCR products of 350 bp, which was the* actin* gene product; however, the expected fragments (600 bp) for LRB7 suggested that it was expressed only in roots of* C. bungeana*.

### 3.4. TIP2 Abundance in Roots, Stems, and Leaves of* C. bungeana*

Then we isolated total proteins from leaves, stems, and roots of* C. bungeana* to measure the abundance of TIP2. To obtain the antibody (anti-LRB7) for western blot, we first confirmed the fusion protein GST-LRB7 in the cell lysates from* E. coli* BL21 (DE3) pLysS transformed with PGEX-4T-3-LRB7. In Figure 3(b). It is clear that there is an additional protein band (approximately 30 KDa) in CK2 (inducted with 20% IPTG), compared to CK1 (without induction). It meant that glutathione-S-transferase (GST) protein was successfully induced in CK2. Next, five cell lysates (lanes 1 to 5) from* E. coli* BL21 (DE3) pLysS transformed by PGEX-4T-3-LRB7 were cultured at 2, 3, 4, 5, and 6 hr. Compared with CK1 and CK2, a protein band of approximately 45 kDa was found in these five lanes revealing that fusion protein GST-LRB7 was expressed successfully. The expression of GST-LRB7 was increased by induction time and reached the maximum at 4 hr. After antibody (anti-LRB7) was purified from the serum of male adult rabbits immunized by GST-LRB7 proteins, it was used for western blot analysis in leaves, stems, and roots of* C. bungeana*, respectively. The antiserum against protein encoded by LRB7 specially recognized 25 kD band only in roots, but not in stems or leaves (Figure 3(c)). Thus, it is deemed that LRB7 gene and its protein (TIP2) were expressed exclusively in roots, similar to TobRB7 gene (S45406) and DcRB7 gene (AB000506).

## 4. Discussion

**Figure 4:**
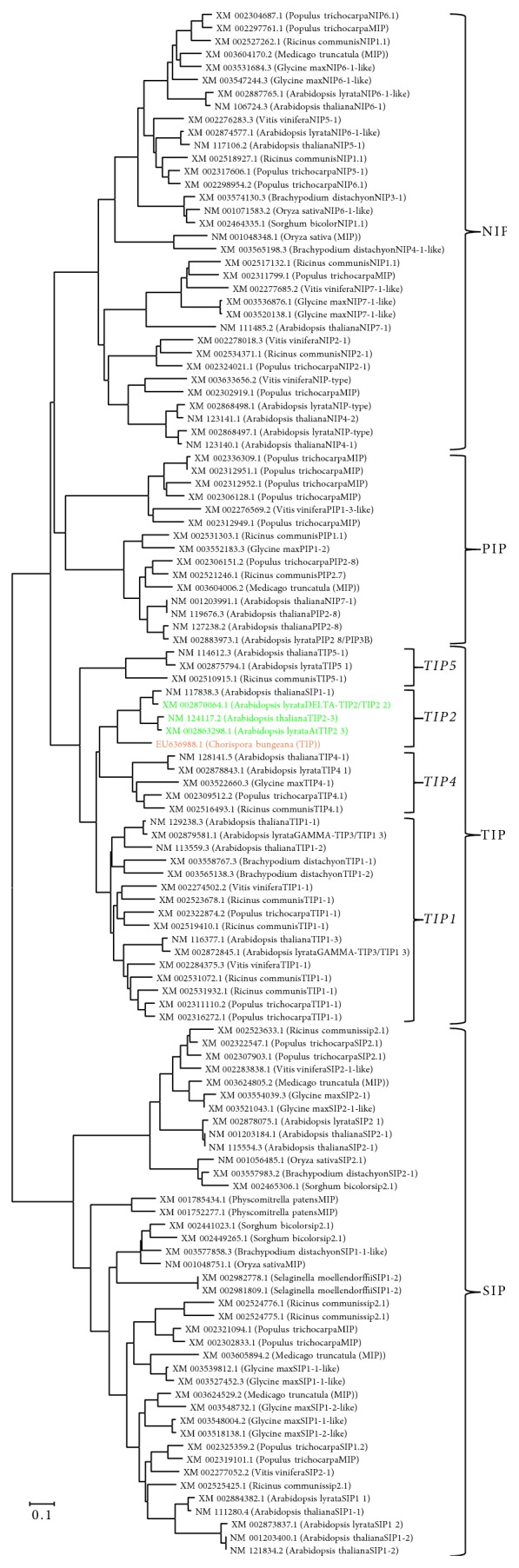
Phylogenetic analysis of mRNAs of MIPs. TIP2 from other species were present in green and LRB7 (EU636988.1) was present in orange. Numbers on the nodes indicate bootstrap values from 1000 replicates and the bar (0.1) represents the scale for branch length.

Aquaporins are membrane channel proteins belonging to the major intrinsic protein (MIP) family and have been characterized in essentially all living organisms [30, 31]. The protein sequences of aquaporins show some highly conserved motifs including six putative transmembrane domains with the N- and C-terminals facing the cytosol, five loops (loops a–e in Figures 2(c) and 2(d)) joining the transmembrane helices, and two NPAs, of which “SGxHxNPAVT” is a maker of aquaporins [25] (Figure 2(d)). Among the five subgroups of MIP, PIPs and TIPs are the most abundant aquaporins in the plasma membrane and tonoplast, respectively [32]. In* Arabidopsis* there are 9 homologs of TIPs which have been characterized and the identity between each other is not high. Besides the phylogenetic tree analysis of protein sequences, further subgrouping of LRB7 encoded protein required the comparison of RNA sequences of MIPs [33]. As shown in Figure 4, the phylogenetic tree of 119 mRNA sequences of MIPs supported LRB7 of* C. bungeana* is TIP2 gene.

TIPs and other aquaporins can be detected in many cellular types of plants. For example, in* Arabidopsis* and* Raphanus* the genes encoding TIP2s are expressed highly in young shoot, rosette, and flower tissues [20, 21]. Although TIP2 has been identified in other plants [34], this is the first time to characterize the full cDNA and protein sequences of TIP2 in* C. bungeana*. Our findings fill the gaps of water channel protein identification of* C. bungeana* and are important to understand the mechanism of flowing water through roots to other tissues in* C. bungeana*. It may help understand the potential functions of aquaporins in chilling-tolerance because* C. bungeana* is an ideal material for investigating the chilling adaptation mechanism of the plant that can survive under frequent temperature fluctuations and freezing temperatures but does not possess special morphological characteristics for preventing chilling damage [18].

The mechanism of chilling-tolerance is tightly linked with redox balance of the cellular redox molecule. In early chilling, the redox transition of ubiquinone can not only ensure the fluency of electron transfer in mitochondria but also facilitate the regulation of the whole-cell redox states leading to adaptation of cellular regulations. Ca^2+^ distribution is a response of* C. bungeana* in adapting to the alpine subnival environment and the accumulation of Ca^2+^ can play an important role in active cold-hardiness [35]. A synergy between antioxidant enzymes such as superoxide dismutase, dehydroascorbate reductase, ascorbate peroxidase, and glutathione reductase leads to a low autoxidation rate that contributes to the protection of the cell membranes and plays an important role in the resistance of suspension cultured cells of* C. bungeana* to sudden freezing [17].

**Figure 5:**
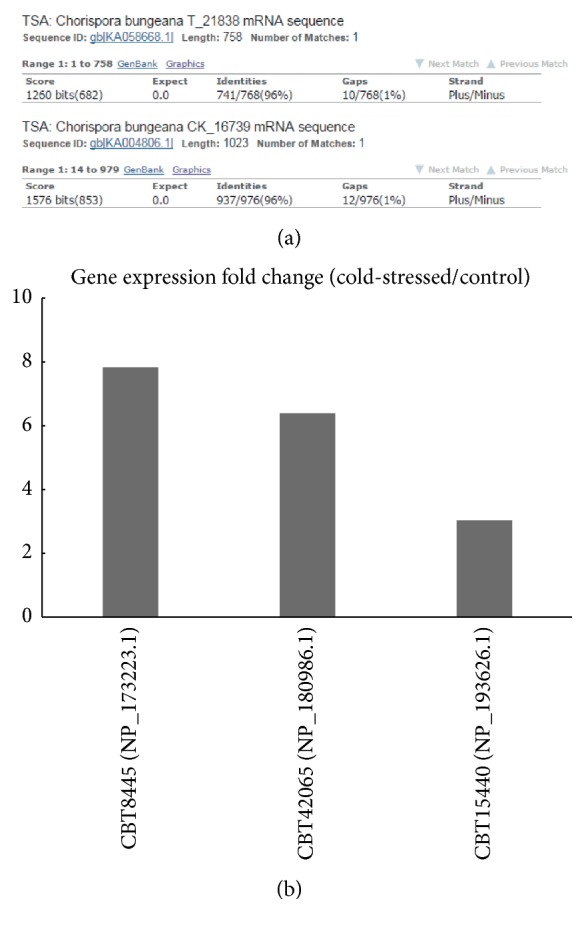
BLAST results of LRB7 gene using TSA database. (a) It showed that LRB7 gene has been assembled in our previous RNA-Seq study. The strands are different because RNA-Seq method used in previous study cannot tell the directions of assembled fragment. (b) Expression patterns (cold-stressed/control) of three genes which can encode water channel proteins.

In rice, a gene called RWC1 encoding 290-residue protein (a water channel protein) is experimented to involve in chilling-tolerance [36]. It is suggesting that water channel protein may affect its downstream cell activities under freezing environment. Linking LRB7 gene and TIP2 identified in this study to our previous RNA-Seq study [19], LRB7 gene has been assembled and detected in both cold-stressed (with the ID of T_21838) and control (with the ID of CK_16739) samples, resulting in 96% and 96% identities (Figure 5(a)). There are three genes whose products could match water channel proteins in other species characterized in the RNA-Seq study, showing the IDs of CBT8445, CBT42065, and CBT15440. Their expression levels are significantly upregulated in the cold-stressed sample, compared to control (Figure 5(b)). In addition, the RNA-Seq study has revealed that the upregulated genes in cold-stressed sample are enriched in the response of water deprivation. Based on these evidences, we implicate that LRB7 is associated with certain chilling-tolerance pathways; however, relationship between water channel proteins and chilling-tolerance should be well studied to attribute to elucidation of the chilling adaptation mechanism in* C. bungeana* by more functional experiments.

## Supplementary Material

Supplementary Figure S1. Phylogenetic analysis of the protein encoded by LRB7 in Chorispora bungeana and MIPs from other species. It revealed the protein encoded by LRB7 was from TIP2 subfamily because it was clustered with TIP2 proteins encoded by A. Thaliana (NP_193465.1 and NP_199556.1) and A. lyrata (XP_002870110.1 and XP_002863344). LRB7 encoded protein and TIP2 proteins encoded by other species were colored in light green.

## Figures and Tables

**Table 1:** DNA/RNA primer sequences used in this study.

Primers	From 5′ to 3′	Denote
P1	TTGCTGGDGTTGGGTCHGC	For the first fragment of LRB7 cDNA
P2	TCCGCGGCYGTKGCRTAAACI	For the first fragment of LRB7 cDNA
P3	CGCTTTGGTCTACACCGTCTA	For the second fragment of LRB7 cDNA
P4	TCGGAAGAACMCAYGAASACAT	For the second fragment of LRB7 cDNA
P5	GCGGAATTCATGGCAGGAGTAGCCTTCGGT	For the genomic region of LRB7
P6	GCGAAGCTTGAAATCAGAAGAA GCAAGAGG	For the genomic region of LRB7
P7	GGAGCTGAGAGATTCCGTTGC	For the actins gene
P8	GAAG CATTTCCTGTGGACAATCGA	For the actins gene
P9	ATCTTCTTCATTAAACAAAAAC	Semiquantitative RT-PCR analysis of LRB7
P10	GTAAACGGTGTAGACCAAA	Semiquantitative RT-PCR analysis of LRB7
5′ GSP1	GAGATGTTGGCTCCGATTGCGAC	For the 5′ RACE of LRB7
5′ GSP2	CTAGTCCGGGCGTGTCAAGAGCA	For the 5′ RACE of LRB7
3′ GSP3	TCACTGGGTCTACTGGGTGGGTC	For the 3′ RACE of LRB7
3′ GSP4	TGCCGGAGCTGTTTATGGCAATG	For the 3′ RACE of LRB7

**Table 2:** MIP sequences used for phylogenetic analysis.

Subgroup	Number	Species
NIP	4	P_trichocarpa; V_vinifera
NIP1	2	R_communis
NIP2	1	V_vinifera
NIP3	1	B_distachyon
NIP4	3	A_thaliana; B_distachyon
NIP5	2	A_thaliana; V_vinifera
NIP6	4	A_thaliana; G_max; P_trichocarpa
NIP7	4	A_thaliana; G_max; V_vinifera
PIP	5	P_trichocarpa
PIP1	3	G_max; V_vinifera; R_communis
PIP2	4	A_thaliana; P_trichocarpa; R_communis; A_lyrata
PIP3	2	A_thaliana
SIP	3	P_trichocarpa
SIP1	13	A_thaliana; G_max; P_trichocarpa; V_vinifera; B_distachyon; A_lyrata
SIP2	13	A_thaliana; G_max; P_trichocarpa; V_vinifera; B_distachyon; R_communis; A_lyrata
TIP	7	R_communis; A_lyrata
TIP1	7	A_thaliana; B_distachyon; V_vinifera
TIP2	6	A_thaliana; P_trichocarpa; A_lyrata
TIP3	3	P_trichocarpa; A_lyrata
TIP4	4	A_thaliana; G_max; P_trichocarpa; A_lyrata
TIP5	2	A_thaliana; A_lyrata
Unknown	26	O_sativa; M_truncatula; S_moellendorffii; R_communis; P_patens; A_lyrata; S_bicolor; P_trichocarpa
